# An interesting case series of ticagrelor induced long QTc

**DOI:** 10.1016/j.ijcrp.2024.200311

**Published:** 2024-07-17

**Authors:** Alireza Farzaei, Entezar Mehrabi Nasab, Yaser Jenab, Alireza Amirzadegan, Alireza Khodayari Javazm, Mokhtar Eisvand, Fateme Hajzeinolabedini, Ali Bozorgi

**Affiliations:** a Tehran Heart Center, Cardiovascular Diseases Research Institute, Tehran University of Medical Science, Tehran, Iran; b Department of Cardiology, School of Medicine, Tehran Heart Center, Tehran University of Medical Sciences, Tehran, Iran; c Department of Cardiology, School of Medicine, Valiasr Hospital, Zanjan University of Medical Sciences, Zanjan, Iran; d Faculty of Medicine, Kermanshah University of Medical Science, Kermanshah, Iran

**Keywords:** Ticagrelor, Long QTc, ACS

## Abstract

This case series explores three patients who underwent percutaneous coronary intervention (PCI) and experienced prolonged QT intervals under treatment with Ticagrelor. The first case was a female who presented with chest pain and received a Xience stent. The second case involved a male patient who received two Xience stents. The third case was that of a male patient with LAD stenosis. All three patients received Ticagrelor and exhibited prolonged QTc intervals on their electrocardiograms (ECGs), which was resolved after switching to Clopidogrel. Thus far, the potential impact of Ticagrelor on QT prolongation has not been adequately addressed in the literature. It is hypothesized that Ticagrelor can block adenosine uptake by red blood cells, which may explain QTc prolongation. The results of this case series indicate that Ticagrelor may prolong QTc intervals. Consequently, it is imperative that clinicians are aware of this previously unlisted side effect and that patients are closely monitored while seeking alternative medications to manage the condition.

## 1 Introduction

Acquired QT interval prolongation is characterized by an increase in the interval between the beginning of the QRS complex and the end of the T wave triggered by extrinsic factors. Long QTc (LQTc) is defined as QTc (QT interval corrected for RR interval) ≥ 470 milliseconds (ms) in men and ≥480 ms in women [1]. Prolonged QTc can lead to torsade de pontes (TdP), especially when QTc exceeds 500 ms. A number of factors can influence the development of acquired QTc prolongation, including female gender, age over 65 years, bradycardia, some structural heart diseases, metabolic disorders, brain injuries, and drugs [1]. Among these, drugs are considered the most common causes of acquired LQT [2], including antiarrhythmics, antimicrobials, antihistamines, and antidepressants as the most common drugs associated with this condition [3]. Ticagrelor (AZD6140) is an oral reversible P2Y12 receptor antagonist that blocks ADP-induced platelet aggregation. Ticagrelor's impact on adenosine concentration is a distinct feature of this agent compared to other P2Y12 antagonists, including Clopidogrel, Cangrelor and Prasugrel [4].

The association between ticagrelor and AV block is well known [5]. Nevertheless, the impact of this drug on QTc interval prolongation remains uncertain, requiring more studies and scientific evidence to be confirmed. In this case series, we report three patients who developed QTc interval prolongation following treatment with ticagrelor.

## 2 Case presentation

### 2.1 Case 1

**Fig. 1:**
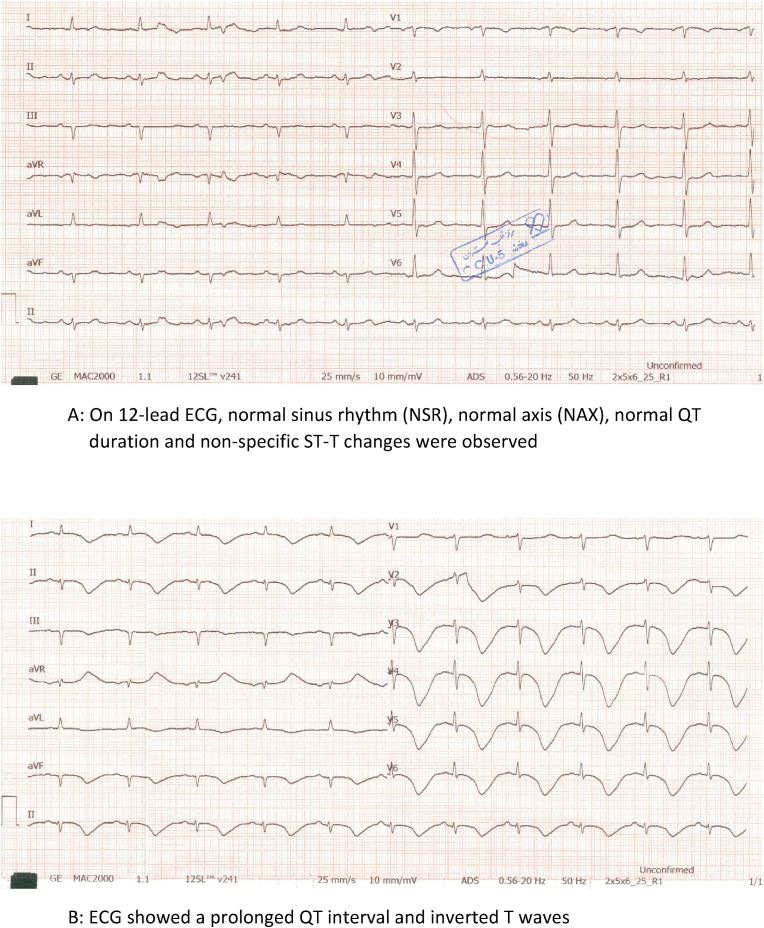
A: On 12-lead ECG, normal sinus rhythm (NSR), normal axis (NAX), normal QT duration and non-specific ST-T changes were observed. B: ECG showed a prolonged QT interval and inverted T waves.

A 72-year-old man visited the emergency department of our hospital complaining of typical chest pain (TCP). He additionally disclosed a history of dyslipidemia (DLP), hypertension (HTN), cigarette smoking and addiction to opium (oral tincture of opium). On 12-lead ECG (Fig. 1A), normal sinus rhythm (NSR), normal axis (NAX), normal QT duration (QTc = 448 ms) and non-specific ST-T changes were observed. Considering the TCP and the presence of risk factors for coronary artery disease, the patient underwent transthoracic echocardiography (TTE), which showed normal LV size, mild LV systolic dysfunction (LVEF = 45 %–50 %), normal RV size and function, no valvular heart disease (VHD) and dilated ascending aorta (42 mm), while no other considerable pathologies were discernible. Coronary angiography (CAG) revealed severe stenosis at the proximal segment of LAD and OM1 arteries, as well as moderate stenosis at the proximal segment of RCA. The patient was scheduled for CABG and after cardiac surgery consultation, the patient preferred PCI over this procedure. Therefore, PCI was performed using two Everolimus-eluting stents (Xience) targeting the LAD and OM1 arteries with a loading dose of 180 mg of Ticagrelor, which was administered immediately before the procedure. Around 36 hours post-PCI, a 12-lead ECG (Fig. 1B) showed a markedly prolonged QT interval and inverted T waves (QTc = 664 ms). Emergency conditions like acute coronary ischemia and intracranial hemorrhage (ICH) were ruled out. Moderately elevated troponin levels, which had been linked to percutaneous coronary intervention (PCI), gradually declined in subsequent readings. Electrolyte levels were measured and found to be normal (Table 1). While the patient was on close cardiac monitoring, an electrophysiologist was consulted with at the first opportunity. Considering that there was no evidence suggesting electrolytic disorders and bradyarrhythmia, the patient's medication history was evaluated by the electrophysiologist (Table 2), raising the suspicion that ticagrelor might be a culprit. Consequently, the aforementioned drug was substituted with Clopidogrel. Serial ECGs showed that the QTc interval declined, reaching 473 ms at one week post-PCI. A 48-h ECG Holter monitoring was performed during wait and watch time, revealing no significant dysrhythmia.

**Table 1:** The laboratory tests of all three patients are listed.

	CASE 1	CASE 2	CASE 3
troponin	positive	positive	negative
Hb	12.5	14.4	13.5
PLT	254000	293000	196000
WBC	6720	10290	6720
K	4.1	3.7	4.7
Na	138	140	135
Ca	9.6	9	9.3
Mg	1.99	2.05	2.26
P	3.2	3.01	3
Cr	1.3	0.78	0.9
FBS	90	86	80
HDL	40	52	48
LDL	145	139	100
TG	135	103	116

**Table 2:** The prescribed drugs to all three patients.

CASE 1	CASE 2	CASE 3
Tab ASA: 300mg stat, 80mg daily	Tab ASA: 300mg stat, 80mg daily	Tab ASA: 300mg stat, 80mg daily
Tab Ticagrelor: 180mg stat, 90mg BD	Tab Ticagrelor: 180mg stat, 90mg BD	Tab Ticagrelor: 180mg stat, 90mg BD
Tab Atorvastatin: 40mg BID	Tab Atorvastatin: 40mg BID	Tab Atorvastatin: 40mg BID
Amp Heparin: 7500–10000 IU/h during PCI	Amp Heparin: 7500–10000 IU/h during PCI	Amp Heparin: 7500–10000 IU/h during PCI
Tab Nitroglycerine long acting: 2.6mg BID	Tab Metoprolol: 47.5mg daily	
Tab Nicorandil: 10mg BID	Tab Amlodipine/Valsartan: 5–80mg daily	

### 2.2 Case 2

**Fig. 2:**
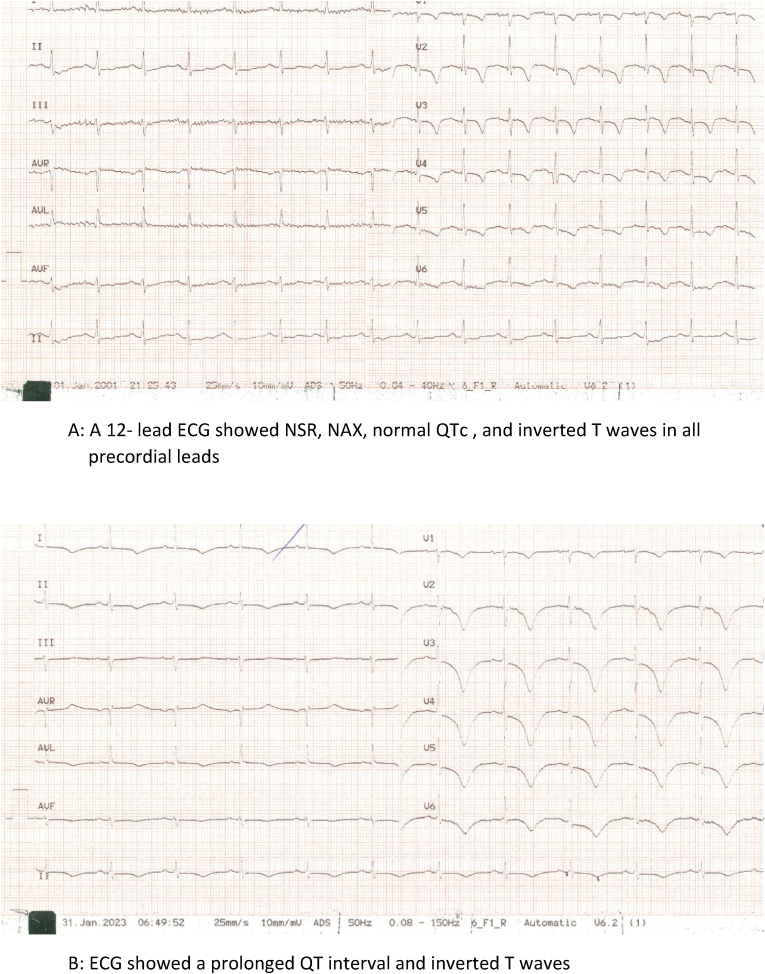
A: A 12- lead ECG showed NSR, NAX, normal QTc, and inverted T waves in all precordial leads. B: ECG showed a prolonged QT interval and inverted T waves.

A 64-year-old woman was presentedto the emergency room complaining of retrosternal chest pain starting a few hours ago. The patient mentioned that she suffered from HTN. On arrival, vital signs were stable and blood pressure was 130/80 mmHg in both arms and the pulse rate was also the same in both hands. The patient was immediately admitted in the emergency room and underwent cardiac monitoring. On admission, ECG (Fig. 2A) showed NSR, NAX, normal QTc (453 ms) and inverted T waves in all precordial leads and TTE demonstrated normal LV size and mild systolic dysfunction (LVEF = 45 %), as well as regional wall motion in the apicoseptal and anteroseptal parts of the LV. The size and function of the RV were normal, and no significant VHD or other notable pathologies were detected. Due to the presence of TCP and dynamic ischemic changes in ECG and echocardiography, the patient was a candidate for CAG. Regarding significant LAD ostium stenosis, a single-vessel disease was suspected. PCI was performed utilizing an Everolimus-eluting Xience stent, preceded by a 180 mg loading dose of Ticagrelor. After PCI, TIMI 3 flow and successful blood flow distal to the stent were achieved, and the patient was transferred to the cardiac care unit (CCU). During the course of heart monitoring, it was observed that the QTc interval exhibited a gradual prolongation, reaching a maximum value of 713 ms (Fig. 2B). Consultation with the electrophysiology service was requested, and the patient's electrolytes were checked immediately checked (Table 1), as well as the list of prescribed medications (Table 2). Considering that there was no evidence for electrolyte disorders and no history of taking a drug with known effects on QTc, the electrophysiologist suggested that the most probable cause could be ticagrelor, so the administration of this drug was stopped, and Clopidogrel was started as replacement. On the next day, QTc started to decline (QTc: 589 ms). The patient was discharged one week later with normal QTc. At the one-month outpatient follow-up, ECG showed a normal QTc interval.

### 2.3 Case 3

**Fig. 3:**
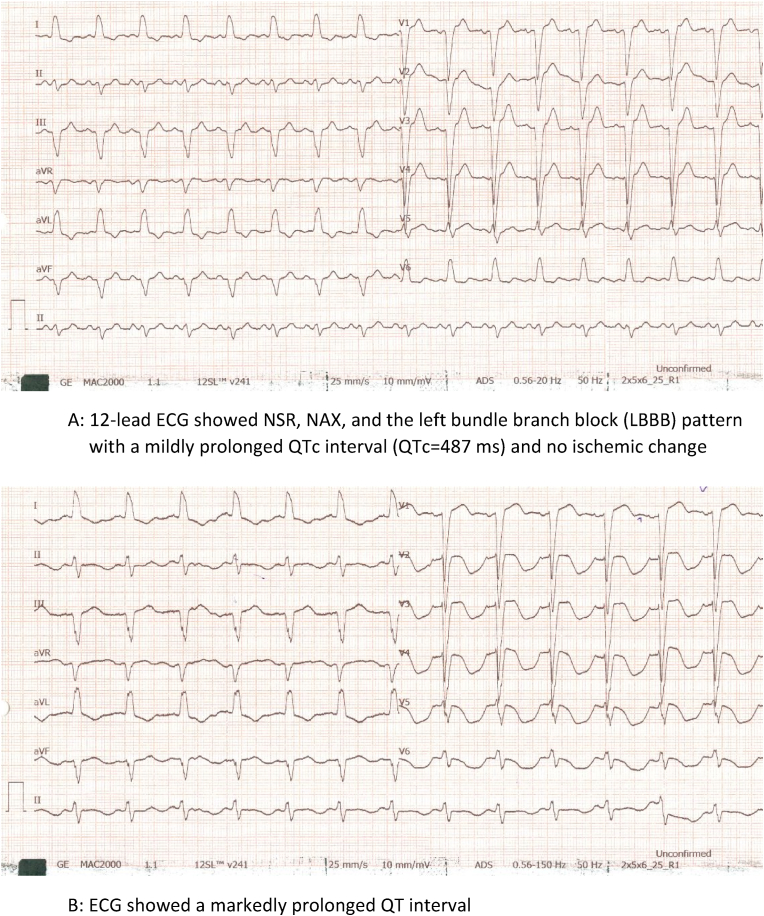
A12-lead ECG showed NSR, NAX, and the left bundle branch block (LBBB) pattern with a mildly prolonged QTc interval (QTc = 487 ms) and no ischemic change. B: ECG showed a markedly prolonged QT interval.

A 70-year-old man with no cardiovascular risk factors complaining of chest pain for the past few months referred to our cardiology clinic. 12-lead ECG (Fig. 3A) showed NSR, NAX, and the left bundle branch block (LBBB) pattern with a mildly prolonged QTc interval (QTc = 487 ms) and no ischemic change. TTE showed mild LV enlargement and moderate systolic dysfunction (LVEF = 40 %) with global dyskinesia, normal RV size and function, and no significant VHD. Coronary CTA (CCTA) revealed severe stenosis at mid LAD, suggesting the patient was a candidate for CAG, which verified mid-part LAD artery stenosis. PCI was conducted using an Everolimus-eluting stent post and the administration of a 180 mg Ticagrelor loading dose. ECG (Fig. 3B) on the day following PCI showed evidence in favor of an increased QTc interval, peaking at 750 ms two days post-procedure. Laboratory tests, including the serum levels of electrolytes, (Table 1), and the list of the patient's medications (Table 2) was checked. There disclosed no electrolyte disorders, and medication history was unremarkable except for Ticagrelor. Consequently, Ticagrelor was ceased and replaced with Clopidogrel. From the evening of the same day, QTc interval gradually decreased and returned to the initial level within a week.

## 3 Discussion

The term Tdp was first introduced by Dessertenne in 1966 for polymorphic ventricular arrhythmia with a pattern of torsade de pointes. Cardiac arrest due to TdP and drug-induced LQT is rare but very catastrophic. The incidence of drug-induced LQT is higher in hospitalized patients than in outpatients because of the presence of other risk factors for proarrhythmic events in hospitalized individuals. Cardiac arrest can be prevented by the timely detection of drug-induced QT interval prolongation and the immediate withdrawal of the culprit drug [5]. TdP often occurs following QT prolongation and T-U wave morphology changes, and QT prolongation is due to the abnormal function of ion channels and proteins involved in the repolarization of ventricular myocytes. These abnormalities may have genetic or acquired etiologies, and the most common acquired form occurs secondary to medications. Drugs that cause TdP often affect the fast current of potassium (IKr), thereby increasing the QT interval [6]. Many reports show that the QT interval shows the longest repolarization in the M-cell region. This physiological intermural dispersion of repolarization does not usually lead to TdP; however, pre-arrhythmic states may result from specific gene mutations or the use of drugs that selectively prolong the action potential in certain layers of the myocardium (usually the M-cell region), leading to increased intermural repolarization gradients, paving the ground for re-entry and subsequent TdP [7].

A number of drugs have been noted to increase the QT interval, and one of them that has received much attention today is ticagrelor. Ticagrelor is an anti-platelet drug, but by inhibiting the absorption of adenosine, it leads to an increase in the concentration of adenosine in plasma. This drug also affects cardiac function by acting on A1 receptors. Ticagrelor has negative chronotropic and dromotropic effects [8] and can adversely affect the conduction system. The PLATO trial showed that this drug was associated with an increase in the rate of ventricular pauses by ≥ 3 seconds. The effects of ticagrelor on the QT interval have been the subject of conflicting data [9]. For example, one study reported that a single dose of ticagrelor had no effect on QT interval [10], but another report indicated a benign prolongation of QTc, which subsided after Ticagrelor discontinuation [11]. The probable impact of Ticagrelor on QT prolongation has not been adequately addressed in prominent medical texts. So, in this reportwe described three patients hospitalized with the diagnosis of acute coronary syndrome (ACS) and treated with ticagrelor following coronary angiography (CAG) and percutaneously coronary stenting (PCI). All three patients had normal QTc intervals before ticagrelor administration, and after receiving ticagrelor, the QTc interval gradually lengthened in all three of them. Subsequently, following the cessation of ticagrelor administration, the QT interval returned to within the normal range.

In a 1994 study, adenosine infusion was reported to result in significant QTc prolongation in patients with coronary artery disease (CAD). Considering that ticagrelor also affects A1 channels, this action may justify the occurrence of LQTc by ticagrelor; however, it is not yet logical to comment on this with certainty, requiring more studies [12]. In a 2007 study [13], it was observed that among 50 patients, all those with early transmural ischemia exhibited prolonged QTc interval, which preceded other clinically accepted indices of transmural ischemia. The majority of cases had a QTc interval in the range of 400–490 ms, with the highest recorded value being 540 ms. A recent investigation conducted in 2023 revealed that during ST-elevation myocardial infarction (STEMI), the QT interval was prone to elongation but to less than 500 ms [14]. All three of our patients had a normal QT interval in the acute phase of ischemia and before PCI. Therefore, in these patients, QT interval prolongation could not be attributed to myocardial ischemia, rather the presence of acute myocardial ischemia could be regarded as a trigger boosting the vulnerability and sensitivity of the channels. Therefore, changes in ion currents following ticagrelor administration could accelerate QT alteration faster than normal. This hypothesis also needs more studies and has not yet been proven. In our patients, the QTc interval was prolonged after ticagrelor administration and returned to the normal range following its withdrawal, suggesting the role of this medication as a cause for this event. More comprehensive reviews and larger studies are required to approve this effect of ticagrelor, as a common antiplatelet drug used for the treatment of acute coronary syndrome (ACS).

A comprehensive investigation did not reveal any electrolyte imbalances, bradycardia, or specific drugs, with the exception of Ticagrelor, that could explain the prolonged QT interval observed in our patients. This prompted us to suspect the effects of ticagrelor on QT prolongation and to discontinue ticagrelor with this suspicion. Finally, we observed that discontinuation of this drug led to improvement and gradual return of QT interval to its original state. Because the role of ticagrelor in prolonging the QT interval has not yet been definitively established, ticagrelor-induced QT prolongation should be considered after excluding important potential causes such as persistent cardiac ischemia, electrolyte abnormalities, and other medications. Ticagrelor is a valuable antiplatelet drug and has an important place in the treatment of ACS. On the other hand, patients with ACS are vulnerable to the side effects of drugs due to myocyte abnormalities at the molecular level during myocardial ischemia. Therefore, when prescribing drugs to these patients, one should act very cautiously to avoid serious complications. During the acute phase of myocardial ischemia, patients are particularly prone to arrhythmias, and LQTc further increases the risk of Tdp and other ventricular arrhythmias in these patients. Therefore, one should be careful in prescribing drugs with potential adverse effects on QTc as well. Ticagrelor is a potent and ideal antiplatelet medication widely used to treat patients with ACS, but there are only some case reports on its potential side effects. More comprehensive studies are needed to confirm the effects of ticagrelor on QT interval changes.

## Ethics approval and consent to participate

Before starting the project, all patients were fully informed about the process and the methods. It was also emphasized that if they do not agree to participate in this study, there will be no shortcomings in the treatment process. Patients were reassured that their personal information would remain secure, as well as adequate information about related costs and no additional costs. In carrying out this project, all relevant ethical considerations including methodology, patient consent and written consent of the patient were fully justified to the study plan of work and conclusions and writing were observed with all cases. The recommended protocols and all the necessary ethics and ethics committee instructions were implemented.

## Consent for publication

Not Applicable.

## Funding

Not Applicable.

## Availability of data and materials

Not Applicable.

## CRediT authorship contribution statement

**Alireza Farzaei:** Software, Methodology, Investigation. **Entezar Mehrabi Nasab:** Validation, Supervision, Software. **Yaser Jenab:** Writing – original draft. **Alireza Amirzadegan:** Methodology. **Alireza Khodayari Javazm:** Formal analysis, Data curation. **Mokhtar Eisvand:** Formal analysis, Data curation. **Fateme Hajzeinolabedini:** Writing – review & editing. **Ali Bozorgi:** Supervision, Project administration.

## Declaration of competing interest

There is no conflict of interest.
